# Effect of X-ray irradiation on hepatocarcinoma cells and erythrocytes in salvaged blood

**DOI:** 10.1038/s41598-017-08405-z

**Published:** 2017-08-11

**Authors:** Feng-Jiang Zhang, Jin-Ting Yang, Li-Hui Tang, Wen-Na Wang, Kai Sun, Yue Ming, Kanhar Ghulam Muhammad, Yin-Fei Zheng, Min Yan

**Affiliations:** 1 Department of Anesthesiology, the Second Affiliated Hospital, Zhejiang University School of Medicine, Hangzhou, 310009 P. R. China; 2 Biomedical Engineering of Zhejiang University, Hangzhou, 310027 P. R. China

## Abstract

The broad clinical acceptance of intraoperative blood salvage and its applications in cancer surgery remain controversial. Until now, a method that can safely eliminate cancer cells while preserving erythrocytes does not exist. Here, we investigated whether X-ray generated from linear accelerator irradiation at a certain dose can kill hepatocarcinoma cells while preserving erythrocytes. HepG2, SK-Hep1 or Huh7 cells were mixed into the aliquots of erythrocytes obtained from healthy volunteers. After the mixed cells were exposed to 30 Gy and 50 Gy X-rays irradiation, the viability, clonogenicity, DNA synthesis and tumorigenicity of the tumor cells were determined by the MTT assay, plate colony formation, 5-ethynyl-2′-deoxyuridine incorporation, and subcutaneous xenograft implantation into immunocompromised mice. The ATP, 2,3-DPG, free Hb, osmotic fragility, blood gas variables in erythrocytes and morphology of erythrocytes at 0 h, 12 h, 24 h, 48 h, 72 h after irradiation were analyzed. X-ray irradiation at 30 Gy effectively inhibited the viability, proliferation, and tumorigenicity of HepG2, SK-Hep1 and Huh7 cells without noticeably damaging the ability of oxygen-carrying, membrane integrity and morphology of erythrocytes. Theses results suggest that X-ray at 30 Gy irradiation might be safe to eliminate hepatocarcinoma cells while preserving erythrocytes in salvaged blood.

## Introduction

Intraoperative blood salvage is an established method that is used to reduce allogeneic blood transfusion and related complications^1^. However, in cancer surgery intraoperative blood salvage has long been considered a contraindication with fear and doubt that free tumor cells might spread and metastasized during the bloodshed in surgery^2^.

Currently, there are two methods that can be used to remove contaminating tumor cells from salvaged blood: leukoreduction filtration (LDF)^3^ and gamma irradiation^4, 5^. However, LDF is limited to the re-transfusion of salvaged blood containing less than 10^7^ cells^6,7]^. There is a concern that during surgery in patients with tumors, ruptures might occur due to the load of tumor cells that go over the capacity of LDF (e.g., more than 2 × 10^7^ /200 ml)^8^. Gamma irradiation at 50 Gy can eliminate tumor cells from intraoperative blood salvage processing at the rate of at least 10 log^4^. In the last 6 years, in Europe 700 or more patients have been subjected to gamma irradiation in 30 different tumor treating centers^4^. However, there are limitations and disadvantages to using gamma irradiation. First, the gamma ray source is typically caesium-137 (^137^Cs) or cobalt-60 (^60^Co). There are security and safety concerns for active irradiation sources. Appropriate measures are necessary to prevent vandals and thieves. Special protection and monitoring are required to ensure staff safety. Second, gamma irradiation is not readily available. Many hospitals do not have blood irradiators and the blood needs to be transported off site to an irradiation center with the expected prolonged turnaround time.

It is well known that X-ray generated from linear accelerator (LINAC) is primarily used to kill tumor cells in cancer patients. Currently LINAC is widely used in radiotherapy departments, and has been successfully implemented in transfusion to irradiate the blood components at cancer centers^9–11^. Studies have shown that there is no significant difference between ^137^Cs gamma irradiation and X-ray irradiation generated from LINAC^10, 12–14^. A minimum dose of 25 Gy is used to prevent transfusion-associated graft-*versus*-host disease (TA-GVHD), to avoid the damage to blood cells, the maximum dose must not exceed the 50 Gy^15^. However, it remains unclear how to eliminate tumor cells that are mixed into salvaged blood using X-ray irradiation.

The current study was carried out to investigate the effect of X-ray irradiation generated from LINAC on eliminating tumor cells in salvaged blood by measuring the viability, clonogenicity, DNA synthesis and tumorigenicity of human hepatocarcinoma cells. In addition, whether X-ray irradiation affects erythrocytes function was determined. This study might be helpful to explore a new, safe and conventional method to aid intraoperative blood salvage application during cancer surgery.

## Results

### X-ray irradiation inhibited the viability of tumor cell lines *in vitro*

**Figure 1:**
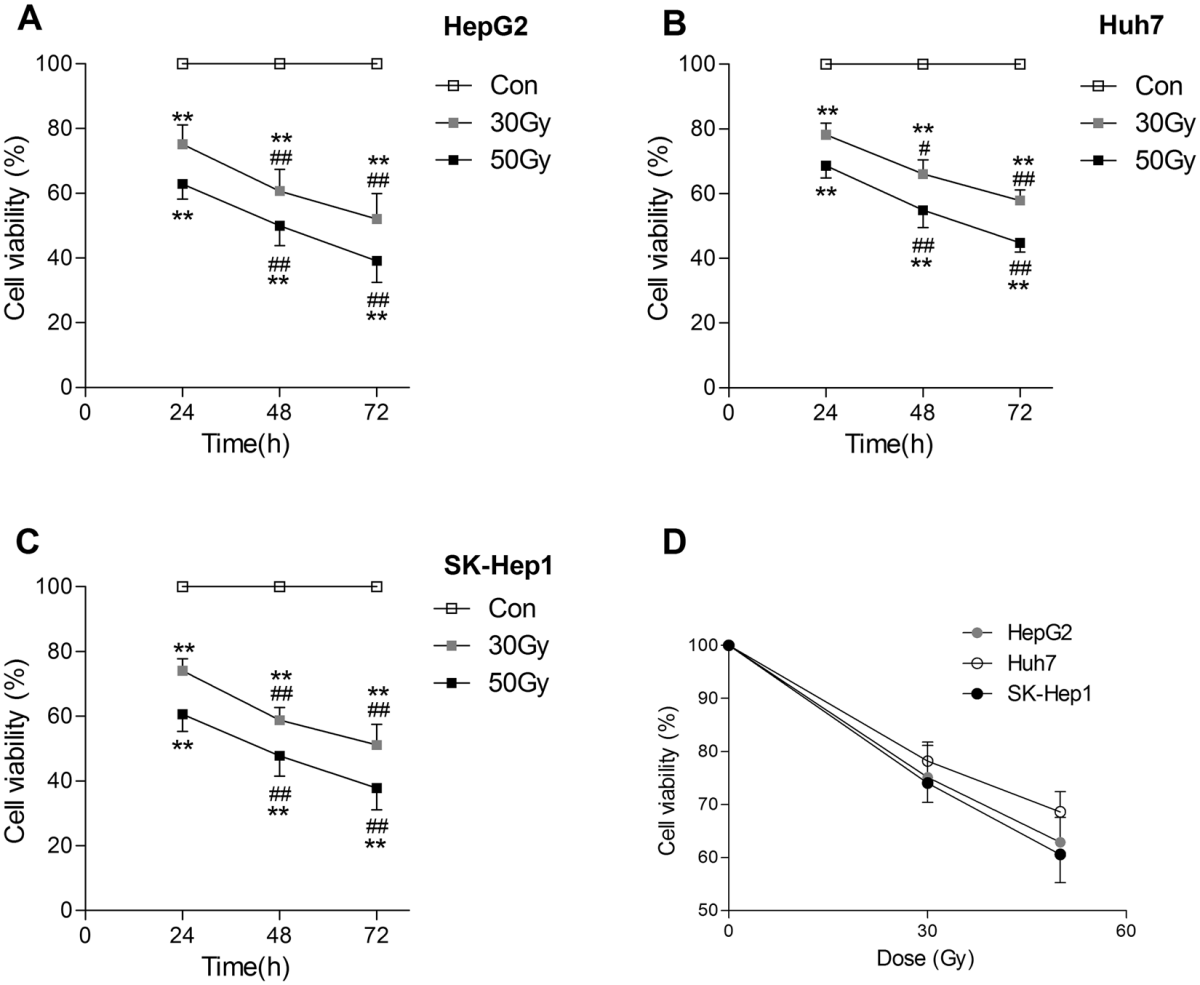
X-ray irradiation inhibited the viability of tumor cell lines *in vitro*. After X-ray irradiation, cell viability was determined using MTT assay in separated HepG2 (**A**), Huh7 (**B**), and SK-Hep1 (**C**) cells after culturing for 24 h, 48 h and 72 h. The cell viability in these three cell lines exposed to 30 Gy and 50 Gy X-rays irradiation after culturing for 24 h is shown in panel D. Date are means ± SEM; *n* = 6. Con: the dose of irradiation was 0 Gy. **p < 0.01 *vs*. the control group at the same culture time; ^#^ *p* < 0.05, ^##^ *p* < 0.01 *vs*. the same treated group cultured for 24 h.

**Figure 2:**
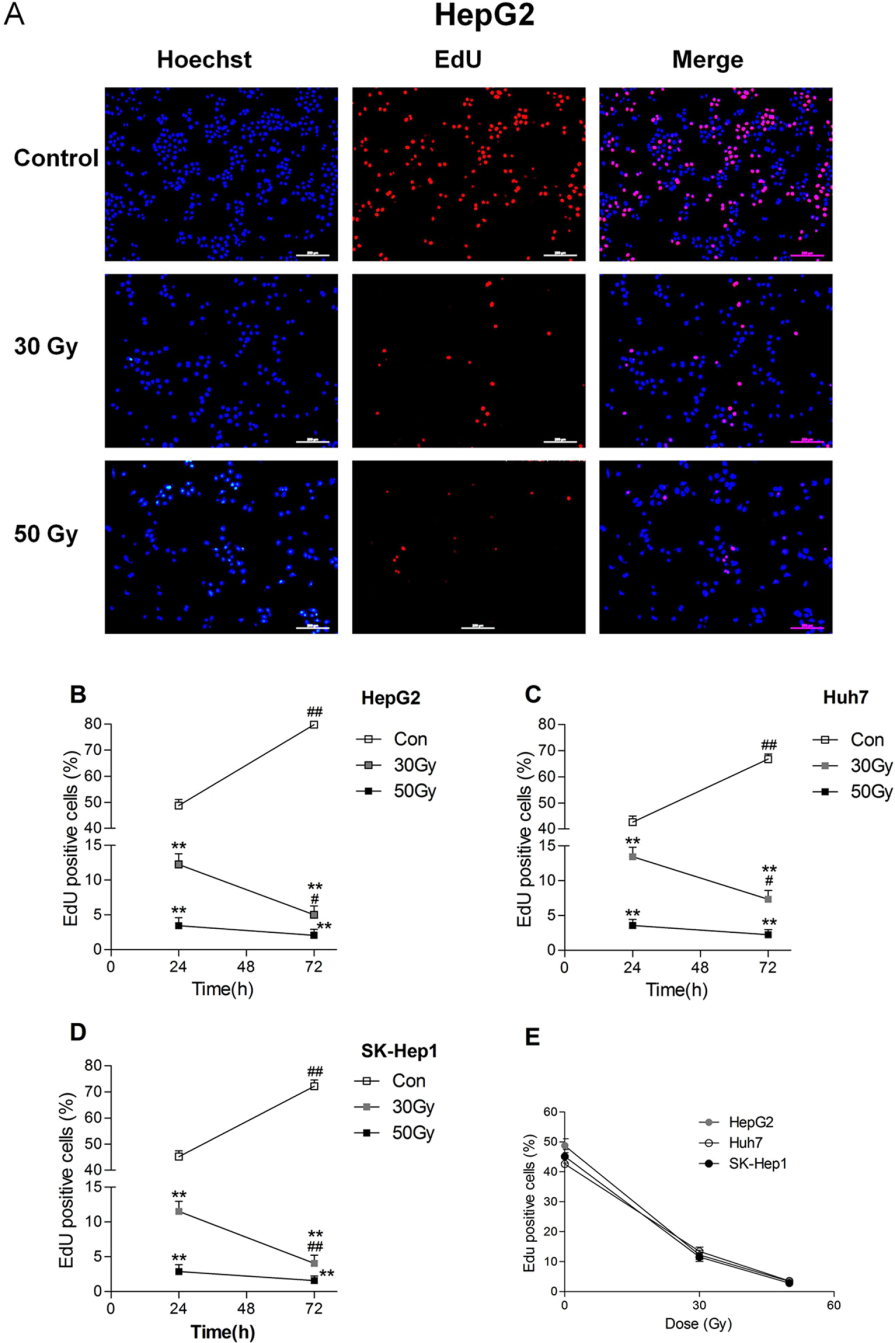
**X**-ray irradiation inhibited DNA synthesis in tumor cell lines *in vitro*. DNA synthesis (scale bar = 200 μm) was detected by 5-ethynyl-2′-deoxyuridine incorporation (EdU) incorporation assay in X-ray irradiated HepG2 cells after culturing for 24 h (**A**). DNA synthesis was detected by EdU incorporation assay in X-ray irradiated HepG2 (**B**), Huh7 (**C**), and SK-Hep1 (**D**) cells after culturing for 24 h and 72 h. DNA synthesis in these three cell lines exposed to 30 Gy and 50 Gy X-ray irradiation after culturing for 24 h is shown in panel E. Date are means ± SEM; *n* = 6. Con: the dose of irradiation was 0 Gy. **p < 0.01 vs. the control group at the same culture time; ^#^ *p* < 0.05, ^##^ *p* < 0.01 vs. the same treated group cultured for 24 h.

**Figure 3:**
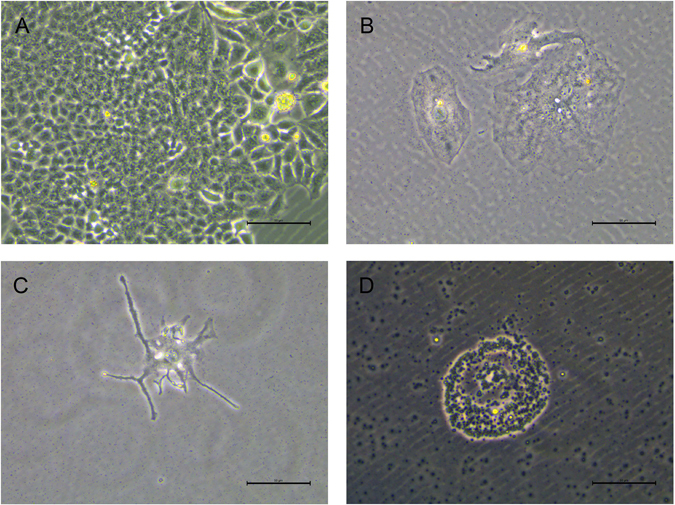
X-ray irradiation induced death in HepG2 cells *in vitro*. After 0 Gy (**A**), 30 Gy (**B**) and 50 Gy (**C**) X-rays irradiation, HepG2 cells were cultured for 7 d. HepG2 cells were cultured for 14 d after exposure to 30 Gy X-ray irradiation (**D**). Scale bar = 50 μm.

The viability (Fig. 1) and DNA synthesis (Fig. 2) in HepG2, Huh7 and SK-Hep1 cell lines were all significantly inhibited by 30 Gy and 50 Gy X-rays irradiation. Cell viability and DNA synthesis were both significantly decreased in all 30 Gy X-ray irradiation-treated tumor cell lines cultured for 72 h compared with those cultured for 24 h (Figs 1 and 2). No colony formation was found in the three tumor cell lines treated with 30 Gy and 50 Gy X-rays irradiation (Fig. S1). The minimal rate of reduction by 30 Gy for the three tumor cell lines, the ratio of the number of treated tumor cells in the assay to the number of untreated cells resulting in colony formation^4^, was more than 3.9 log. Cellular swelling, membrane rupture, and cytoplasm leakage were found in different dose of X-ray irradiation-treated HepG2 cells after culturing for 7 d (Fig. 3A–C). The X-ray irradiation-treated HepG2 cells were totally lysed after culturing for 14 d (Fig. 3D). The similar morphological damages were found in X-ray irradiation-treated Huh7 and SK-Hep1 cells after culturing for 7 d and 14 d (data not shown).

### X-ray irradiation *in vitro* inhibited the growth of xenograft tumors in immunocompromised mice

**Figure 4:**
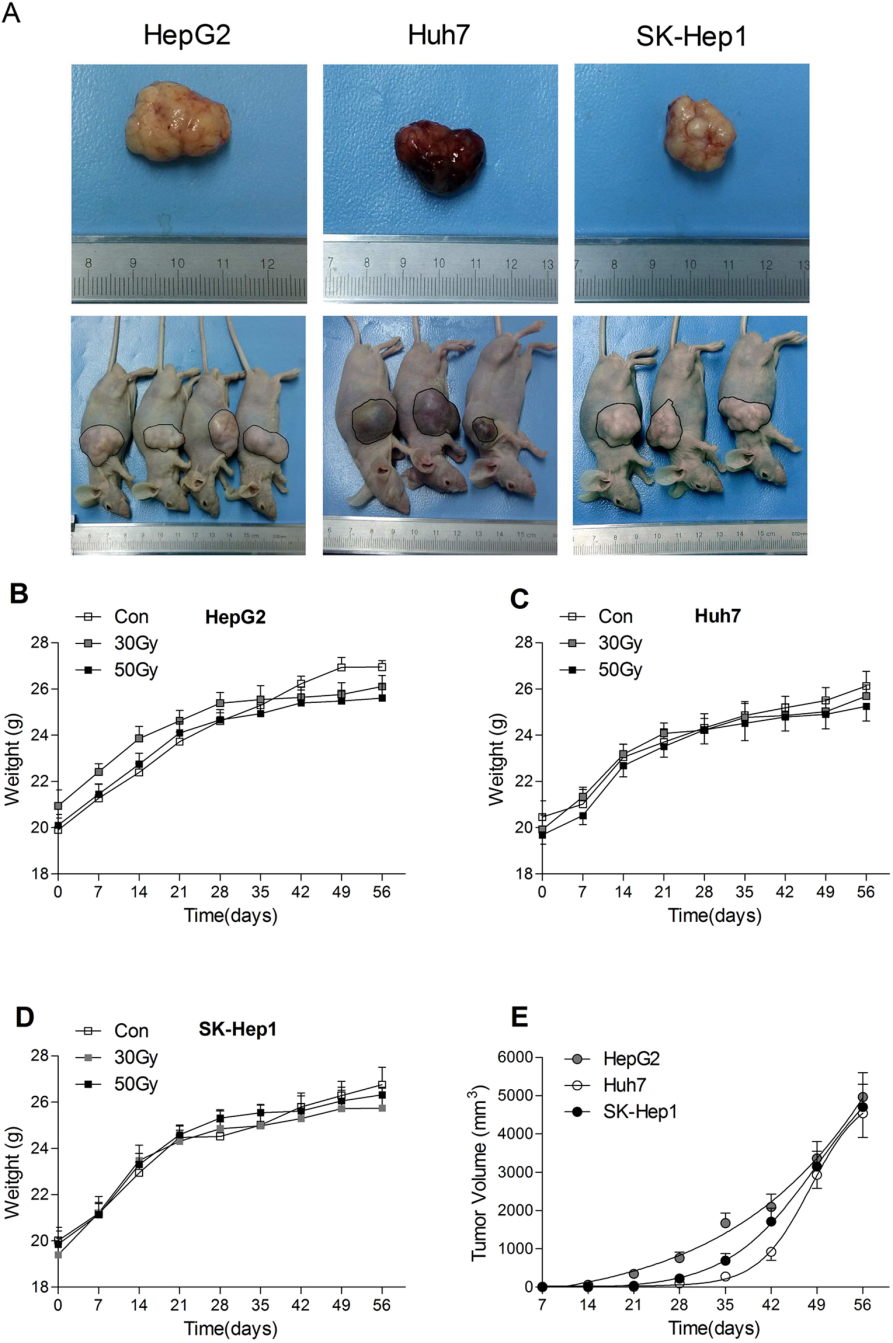
X-ray irradiation inhibited the growth of xenograft tumors in immunocompromised mice. The subcutaneous xenograft tumors developed by non-irradiated HepG2, Huh7 and SK-Hep1 cells in immunocompromised mice (**A**). The body weights of immunocompromised mice subcutaneously xenotransplanted with HepG2 (**B**), Huh7 (**C**) and SK-Hep1 (**D**) cells. The volume of xenograft tumors in immunocompromised mice subcutaneously xenotransplanted with non-irradiated tumor cells (**E**). Date are means ± SEM; *n* = 8 mice in each group.

All the subcutaneous xenotransplantation of non-irradiated HepG2, Huh7 and SK-Hep1 cells into immunocompromised mice resulted in xenograft tumors (8 mice for each tumor cell line) (Fig. 4A). The volume of xenograft tumors in immunocompromised mice subcutaneously xenotransplanted with non-irradiated tumor cells was developed in a time-dependent manner (Fig. 4E). There was no xenograft tumor progress in any of the 48 immunocompromised mice subcutaneously xenotransplanted with X-ray (30 Gy and 50 Gy) treated HepG2, Huh7 and SK-Hep1 cells (8 mice in each group). The body weights of immunocompromised mice increased in a time-dependent manner after xenotransplantation with tumor cell lines, and there was no significant difference of body weights between the control group and irradiated groups (Fig. 4B–D).

### Effects of X-ray irradiation on erythrocytes *in vitro*

**Figure 5:**
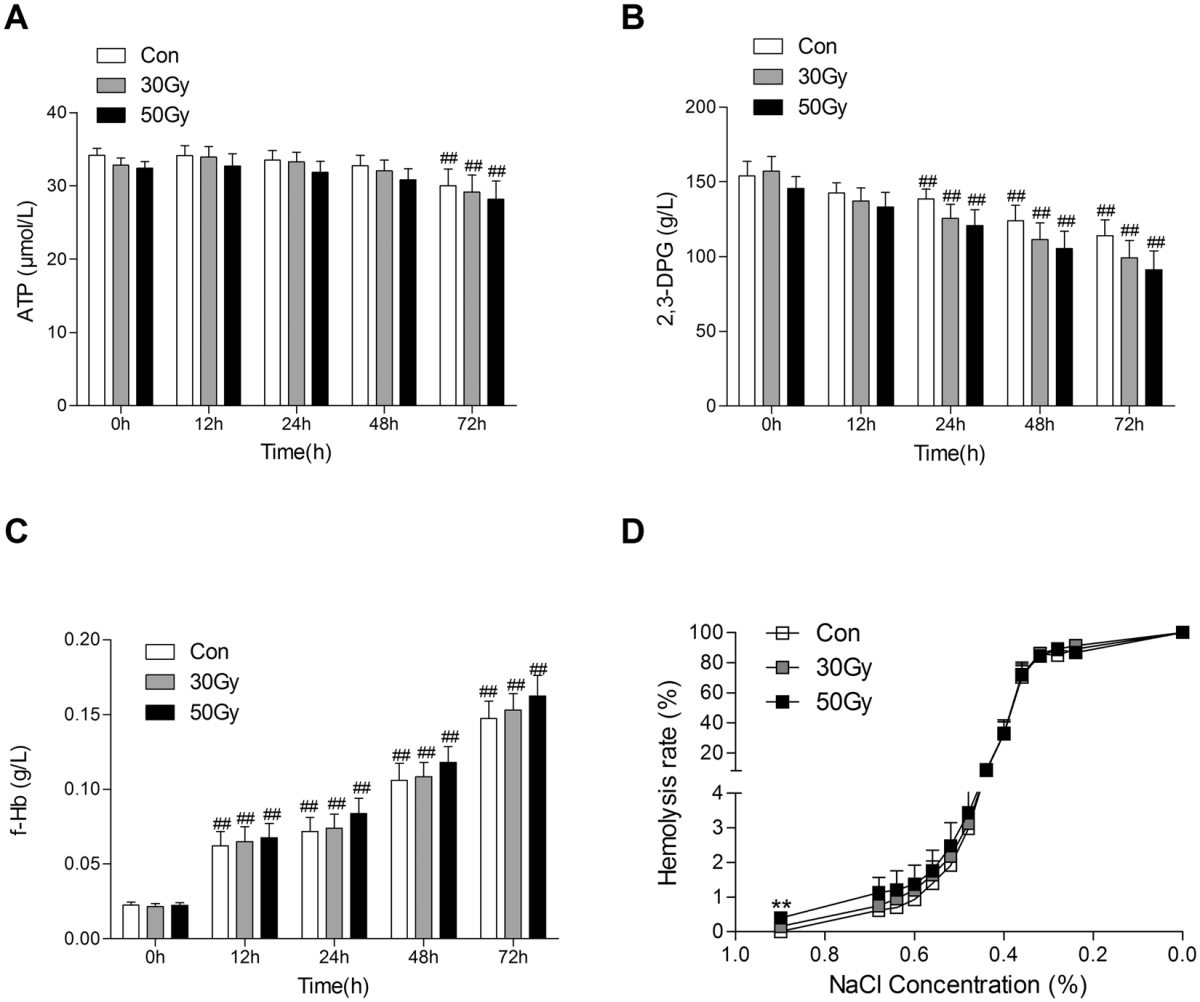
Effects of X-ray irradiation on erythrocytes *in vitro*. ATP (**A**), 2,3-DPG (**B**), and free Hb (**C**) were detected in X-ray irradiated erythrocytes after culturing for 0 h, 12 h, 24 h, 48 h and 72 h. Osmotic fragility (**D**) was assayed in X-ray irradiated erythrocytes after culturing for 0 h. Data are means ± SEM; *n* = 14. ^**^ *p* < 0.01 *vs*. Con (0 Gy at 0 h after irradiation).

ATP were obviously reduced in erythrocytes cultured for 72 h, 2,3-DPG were significantly reduced for 24 h to 72 h, and free Hb was significantly increased 12 h to72 h compared with those cultured for 0 h regardless of X-ray treatment (Fig. 5A–C). Although theses parameters in erythrocytes were slightly altered by X-ray irradiation in a dose dependent manner, there was no significant difference among all groups in the same culturing time point (Fig. 5A–C). Furthermore, the echinocyte shape transformation was increased in erythrocytes during the culturing period regardless of X-ray treatment, but there was no difference among all groups in the same culturing time point (Fig. S2). The morphologic alterations in erythrocytes might be due to ATP declining and loss of phospholipid during storage or culturing^16, 17^. Culturing itself can damage erythrocytes and the intraoperative salvaged erythrocyte is recommended to be transfused immediately or within 6 h^18^. Moreover, Many studies suggested irradiation did not obviously influence the function of erythrocytes^4, 9, 10, 15^. Therefore, we focused on the effect of X-ray irradiation on tumor cells and erythrocytes without culturing. All blood gas variables, including extracellular K^+^ and Na^+^, pH, PO_2_, P_50_, Hb and Cl^−^ in erythrocytes, were not significantly affected by 30 Gy or 50 Gy X-ray irradiation (*p* > 0.05 *vs*. the control group, Table 1). It is worth noting that osmotic fragility in erythrocytes was significantly increased by 50 Gy X-ray irradiation, but not by 30 Gy X-ray irradiationin compared with the control group (*p* < 0.01, Fig. 5D).

**Table 1:** Effects of X-ray irradiation on blood gas variables in erythrocytes *in vitro*.

	Con	30 Gy	*p*-value	50 Gy	*p*-value
Hb (g/L)	138.48 ± 10.33	135.45 ± 10.49	0.906	134.18 ± 10.76	0.867
pH	7.47 ± 0.06	7.46 ± 0.06	0.939	7.45 ± 0.06	0.891
P_50_ (mmHg)	14.89 ± 1.90	16.04 ± 2.00	0.611	16.58 ± 2.07	0.457
PO_2_ (mmHg)	149.39 ± 5.00	156.05 ± 5.32	0.656	158.63 ± 4.44	0.538
K^+^ (mmol/L)	3.94 ± 0.33	4.26 ± 0.34	0.546	4.87 ± 0.44	0.091
Na^+^ (mmol/L)	144.69 ± 1.33	143.93 ± 1.58	0.708	143.31 ± 1.34	0.500
Cl^−^ (mmol/L)	136.59 ± 1.24	136.84 ± 1.21	0.838	137.08 ± 1.30	0.790

Blood gas variables were detected in X-ray (0, 30 and 50 Gy) irradiated erythrocytes after culturing for 0 h. Data are presented as means ± SEM, *n* = 8. Con: the dose of irradiation was 0 Gy. There was no significant difference among the three groups. Hb: hemoglobin; pH: potential of hydrogen; P_50_: partial pressure of oxygen at which hemoglobin is half-saturated with oxygen; PO_2_: partial pressure of oxygen; K^+^: serum potassium ion; Na^+^: serum sodium ion; Cl^−^: serum chloride ion.

## Discussion

Primary liver cancer is the fifth most commonly diagnosed cancer in men and the second leading cause of cancer death worldwide^19^. Approximately 75% of the total liver cancer occurs in Asia, with China alone accounting for more than 50% of the globe^19^. Surgical ablation and orthotopic liver transplantation continue to be the most popular therapeutic options for various liver cancers. Liver surgery and transplantation are associated with massive intraoperative hemorrhage and frequently require large amounts of allogeneic blood transfusion^8, 20^. However, allogeneic blood shortage is widespread^21^ and is particularly critical in China^22^.

Our study showed that the cell viability, DNA synthesis, and colony forming ability of hepatocarcinoma cells mixed in salvaged blood (5 × 10^5^ /ml cell lines in erythrocytes) were inhibited by X-ray irradiation in a dose-dependent manner. The colony formation of all three cell lines was completely inhibited by 30 Gy and 50 Gy X-rays irradiation. It was further confirmed by the xenograft tumor model in immunocompromised mice, in which the tumorigenicity of all three hepatocarcinoma tumor cell lines was entirely inhibited by 30 Gy and 50 Gy X-rays irradiation. The most important molecular target of irradiation is eukaryotic DNA, so it is unimpaired for anucleated erythrocytes in theory. In our study 30 Gy X-ray irradiation did not significantly influence the membrane integrity, osmotic fragility, or oxygen-carrying function of erythrocytes. Free Hb, pH, P_50_, PO_2_, extra-erythrocytic K^+^ and Na^+^ in erythrocytes were not considerably affected by 30 and 50 Gy irradiation. The ATP, 2,3-DPG, and free Hb were not significantly altered in all X-ray irradiated erythrocytes compared with the non-irradiated erythrocytes even culturing for 72 h. These results were consistent with the recent study that treatment with Cobalt-60 gamma ray irradiation at 20 Gy, 40 Gy, and 60 Gy did not significantly increase the hemolysis in erythrocytes even stored for 1 d to 3 d^23^. Although the echinocyte shape transformation was increased in erythrocytes during the culturing period regardless of X-ray treatment, there was no difference among all groups in the same culturing time point. It is suggested that the intraoperative salvaged erythrocytes should be transfused immediately or within 6 hour^18^ in case of damaging the morphology of erythrocytes due to large losses of ATP and phospholipid during storage or culturing^16, 17^. These results in our current study indicated that eliminating hepatocarcinoma cells without obviously damaging erythrocytes in the intraoperative salvaged blood is feasible. It is worth noting that the osmotic fragility of erythrocytes was significantly increased by 50 Gy X-ray, but not by 30 Gy X-ray irradiation, which might be the reason that excess irradiation induces significantly deformed erythrocytes^23^. Therefore, 30 Gy X-ray irradiation might be used as a safe and effectual technique to inactivate the liver cancer cells (especially in inhibiting tumorigenicity) such as HepG2, Huh7 and SK-Hep1 in the salvaged blood.

After irradiation, necrosis played a key role in cell death. These findings were consistent with those of our previous reports that tumor cells showed necrosis after gamma-ray irradiation^5^. Necrosis in these three tumor cell lines caused by irradiation may be attributed to mitotic catastrophe due to DNA destruction, chromosomal aberrations and dysfunction of cell cycle checkpoints^24^.

The effects on the erythrocytes by X-ray irradiation were similar to gamma irradiation in our previous study^5^. However, the X-ray irradiation was more efficient than gamma irradiation in inhibiting the colony formation of HepG2 cells *in vitro*. The minimal rate of reduction by 30 Gy X-ray irradiation was more than 3.9 log for HepG2 cells, but the gamma irradiation was 2.5 logs. Moreover, X-ray irradiation generated from LINAC, which does not always produce radiation, shows better quality dosimetry, and safer radiation without an active source than gamma radiation. Another advantage of using X-ray irradiation generated from LINAC is that it significantly lowers the cost and time burden *versus* gamma irradiation because LINAC can be easily got from the radiotherapy department in hospitals worldwide^9^.

In conclusion, it has been demonstrated that 30 Gy X-ray irradiation generated from LINAC is a safe and effective method to inactivate HepG2, Huh7 and SK-Hep1 cells while preserving the viability of erythrocytes in the salvaged blood. This X-ray irradiation procedure might help to safely introduce intraoperative blood salvage into cancer surgery, however more *in vivo* studies are needed to evaluate its safety and validity.

## Materials and Methods

### Ethical statement

All methods used in the human studies described were conducted according to the Declaration of Helsinki (Washington 2002) and was approved by the Ethics Committee of 2^nd^ Affiliated Hospital, Zhejiang University School of Medicine.

All protocols and procedures for the experiment using mice were approved by the Ethics Committee for Animal Experimentation of Zhejiang University and were performed according to the Guidelines for Animal Experimentation of Zhejiang University and the National Institutes of Health Guide for Care and Use of Laboratory Animals (NIH Publications No. 80-23) revised in 1996.

### Human study

From April 25, 2013 to May 5, 2015, fourteen healthy Chinese volunteers aged 18 to 45 years, whose body mass index (BMI) was 18.5–24 kg/m^2^ and body weight was higher than 50 kg, were screened and enrolled. The indexes of routine hematology and biochemistry examinations were within the normal range, and the serology for HIV, hepatitis B virus (HBV) and hepatitis C virus (HCV) were negative in all volunteers. The ones suffered cancers and hematologic diseases (anemia and erythrocytes abnormality) were excluded. During this study, no volunteer suffered any cancer or hematologic disease. The study was approved by the Ethics Committee of 2^nd^ Affiliated Hospital, Zhejiang University School of Medicine, on April 22^nd^, 2013. All participants provided written informed consent. The blood of all 14 healthy volunteers was mixed with 200 ml of heparinized normal saline at a concentration of 30 IU/ml. The erythrocytes (250–300 ml) were collected and prepared using a Cell Saver 5 (Haemonetics Co, Boston, MA, USA). The hematocrit of concentrated erythrocytes was 40–60%.

### Study design

#### Protocol 1: Assessement of the Effect of X-ray irradiation on hepatocarcinoma cells in salvaged blood

For this study we sampled erythrocytes from 8 volunteers to use in subcutaneous xenograft experiments *in vivo*, and 6 erythrocyte samples from 6 volunteers were used in experiments *in vitro*. The sampled erythrocytes from 14 volunteers were washed using Cell Saver 5, and each erythrocyte sample taken from 14 volunteers was divided into 3 aliquots that were mixed with HepG2, Huh7 or SK-Hep1 cells. The tumor cell final concentration was 5 × 10^5^ /ml in each aliquot. A single batch of each cell line was subdivided and mixed with the 14 different erythrocyte samples. Three different groups were allocated for each portion of mixed cells: the control group (non-irradiated group), 30 Gy and 50 Gy X-rays irradiation groups. We irradiated all mixed cells with 6 MV LINAC (Varian Trilogy, USA). When the irradiation process was completed, we used single-step density gradient centrifugation in Percoll (GE Healthcare Bio-Sciences AB, Uppsala, Sweden), as described in our previous study^5^. Next, the viability of the separated tumor cells was determined by the MTT assay, plate colony formation, EdU incorporation assay, and subcutaneous xenografting into immunocompromised mice. For xenograft tumor experiments, 72 male immunocompromised BALB/c nude mice were randomly divided into 3 groups xenotransplanted with 0, 30 and 50 Gy X-rays irradiated HepG2 cells, Huh7 and SK-Hep1 cells (*n* = 8).

#### Protocol 2: Assessement of the Effect of X-ray irradiation on erythrocytes in salvaged blood

Erythrocytes collected from 14 volunteers were used in this experiment. After washed by the Cell Saver 5, the erythrocyte sample was divided into 3 aliquots that were mixed with HepG2, Huh7 or SK-Hep1 cells. As described in Protocol 1, the mixed cells consisting of erythrocytes and tumor cells, divided into three groups: the control group (non-irradiated group), 30 Gy and 50 Gy X-rays irradiation groups using LINAC. When the irradiation process was completed, we separated tumor cells and erythrocytes. Then, ATP, 2,3-DPG, free Hb concentration, osmotic fragility and blood gas variables in separated erythrocytes were assayed immediately as previously described^5^. The morphology, ATP, 2,3-DPG, free Hb concentration of separated erythrocytes were assayed after culturing for 12 h, 24 h, 48 h, and 72 as described in the previous study^25^.

### Design of a blood irradiation container and set-up

According to a previous study^9^, a blood irradiator box composed of polymethylmethacrylate (PMMA) with the dimensions of 24 × 24 × 5.5 cm^3^ was used. The thickness of the box walls and top layer was 1 cm, while the bottom layer was 0.5 cm, to guarantee an appropriate build-up of 6 MV. The distance from the source to the surface of the box is about 60 cm. Only one 6 MV direct field of 40 × 40 cm^2^ was at the isocenter.

### Animals

Seventy-two SPF male BALB/c nude mice (18–20 g, six weeks old) were purchased from Shanghai Laboratory Animal Center, Chinese Academy of Sciences (Shanghai, China). Mice were housed in sterile and static micro-isolation cages to be fed on irradiated standard pellet chow and sterile water *ad libitum* in a 12-hour light/dark cycle and a room temperature of 22 ± 1 °C. All cages contained sterile wood shavings, bedding and a cardboard tube for environmental enrichment. All mice were acclimatized for one week before the experiment.

### Tumor cell lines

Human hepatoma cell lines (HepG2, Huh7 and SK-Hep1) were bought from the cell bank of the Shanghai Branch of the Chinese Academy of Sciences. All cell lines were cultured in the Dulbecco’s modified EagIe’s medium (DMEM) containing 10% fetal bovine serum (FBS), 100 units/ml penicillin and 100 mg/ml streptomycin. Cells were incubated at 37 °C in a humidified incubator with 5% CO_2_. All media and FBS were purchased from Gibco (Grand Island, NY, USA).

### MTT assay

Each group of isolated tumor cells was seeded onto 96-well plate (1 × 10^3^ /well for 24 h and 48 h; or 5 × 10^2^ /well for 72 h) in quintuplicate at 37 °C in a humidified incubator containing 5% CO_2_. The cell viability was evaluated by the amount of viable cells stained by 3-(4,5-dimethylthiazol-2-yl)-2,5-diphenyltetrazolium bromide (MTT, Sigma-Aldrich Inc, St Louis, MO, USA), which was released with dimethylsulfoxide (DMSO, Sigma-Aldrich Inc, St Louis, MO, USA). The optical density was detected at 490 nm using an automatic microplate reader (BioRad, Hercules, CA, USA). The irradiation effect that killed the tumor cell lines was calculated as the cell viability, which was calculated as follows: (the percentage of viable cells) = (absorbance of treated wells - absorbance of blank wells)/(absorbance of the control wells - absorbance of blank wells) × 100%.

### Colony formation assay

In this process each of the isolated tumor cells groups was seeded. The control group was seeded onto 6-well plates in triplicate at a density of 2 × 10^2^ /well, and the irradiated groups were seeded similarly at a density of 10^4^ /well and for the medium was exchanged every 2 days. After 14 days of incubation, cells were fixed with methanol and stained with Giemsa. To calculate the colony formation rate the number of colonies was divided by the number of seeded cells^26^.

### EdU incorporation assay

Each group of isolated tumor cells was seeded onto 96-well plates in triplicate at a density of 1 × 10^3^ /well for 24 h of incubation or 5 × 10^2^ /well for 72 h of incubation. The cells were incubated for an additional 2 h in medium containing 50 μM EdU (RiboBio, Guangzhou, China). Cells were then washed with PBS, fixed and permeabilized with PBS containing 4% paraformaldehyde and 0.5% Triton X-100. Cells were then incubated with 1 ×  Apollo reaction cocktail (100 μl/well) for 30 min. DNA was incubated with Hoechst 33342 stain (100 μl/well) for 30 min and was visualized with an inverted fluorescence microscope (Leica DM5500, Germany). For each EdU experiment, five random fields were imaged by 100× magnification. Captured images were processed and analyzed with ImageJ software. The number of EdU positive cells was identified by Hoechst nuclei staining and was expressed as a percentage of the total number of cells in each field^27^.

### Xenograft tumor models

The immunocompromised mice were anesthetized with 50 mg/kg sodium pentobarbital and were xenotransplanted with 2 × 10^6^ cells (HepG2, Huh7 and SK-Hep1) in 200 μl in the left flank near the upper extremity^26^. The tumor volume (*V*) was calculated weekly by measurement (caliper rule) of the tumor length and width according to the formula *V* = π/6 × a × b^2^ (a > b). After 8 weeks, the mice were euthanized by CO_2_, and xenograft tumors were excised to measure the final dimensions and weights. The tumor tissues were then fixed for hematoxylin and eosin (HE) staining.

### Assay of ATP, 2,3-DPG and free Hb concentrations

After irradiation, the free Hb concentration in the solution of erythrocytes and ATP and 2,3-diphosphoglycerate (2,3-DPG) concentrations in the erythrocytes were measured using Quantitative Human Competitive ELISA kits (Hermes Criterion Biotechnology, Canada) according to the manufacturer’s instructions.

### Osmotic fragility test

The osmotic fragility of erythrocytes was studied according to an earlier study^28^. Immediately after irradiation, NaCl ranging from 0.24% to 0.68% at the final concentration was used to incubate the separated erythrocytes. To specify the hemoglobin concentration, the supernatant absorbance at 540 nm was measured with an ultraviolet spectrophotometer. Those erythrocytes that were preserved with normal saline were used as a negative control (0% hemolysis), and those that were preserved with distilled water were used as a positive control (100% hemolysis).

### Assay of blood gas variables

Immediately after irradiation, the extracellular K^+^ and Na^+^ concentration, pH, Hb, PO_2_ (partial pressure of oxygen dissolved in the solution), and P_50_ (the PO_2_ at which hemoglobin is half-saturated with oxygen) values in the erythrocyte solution were measured using an automated blood gas analyzer (Roche Diagnostics Cobas b123, Mannheim, Germany).

### Erythrocyte morphology

The morphology of erythrocytes was examined under an inverted microscopy (Olympus, CKX41, Japan). Samples were diluted 10-fold, and 5 μl of diluted erythrocytes was smeared on glass slides^25^.

### Statistical analysis

The data are shown as means ± SEM. One-way ANOVA was used to analyze the Osmotic fragility and blood gas variables following Student-Newman-Keuls multiple comparison tests. Colony formation, ATP, 2,3-DPG, free Hb concentrations, EdU assay, tumor volume and the weight of immunocompromised mice were analyzed by two-way ANOVA following Student-Newman-Keuls multiple comparison tests. We used GraphPad Prism Version 5.0 (GraphPad Prism Software, San Diego, CA, USA) to acquire all statistical analyses. We determined statistical significance of value *p* < 0.05.

## Electronic supplementary material

Supplementary Information
