# Supplementary material for: Effect of X-ray irradiation on hepatocarcinoma cells and erythrocytes in salvaged blood
Source: Sci Rep. 2017 Aug 11;7:7995. doi: 10.1038/s41598-017-08405-z (PMC5554194; doi:10.1038/s41598-017-08405-z)
Supplement: Supplementary file 1 — Supplementary Information [file 41598_2017_8405_MOESM1_ESM.pdf]

Supporting Information for

Effect of X-ray irradiation on hepatocarcinoma cells and erythrocytes in salvaged blood

Author list and affiliations

Feng-Jiang Zhang[1,+], Jin-Ting Yang[1,+], Li-Hui Tang[1], Wen-Na Wang[1], Kai Sun[1], Yue Ming [1], Kanhar Ghulam Muhammad[2], Yin-Fei Zheng[2] & Min Yan[1,*]

[1]Department of Anesthesiology, the Second Affiliated Hospital, Zhejiang University School of Medicine, Hangzhou 310009, P. R. China.

[2]Biomedical Engineering of Zhejiang University, Hangzhou 310027, P. R. China.

[*]Correspondence and requests for materials should be addressed to M.Y. (email: zryanmin@zju.edu.cn).

[+]These authors contributed equally to this work.

Supplementary Figures 1 – 2

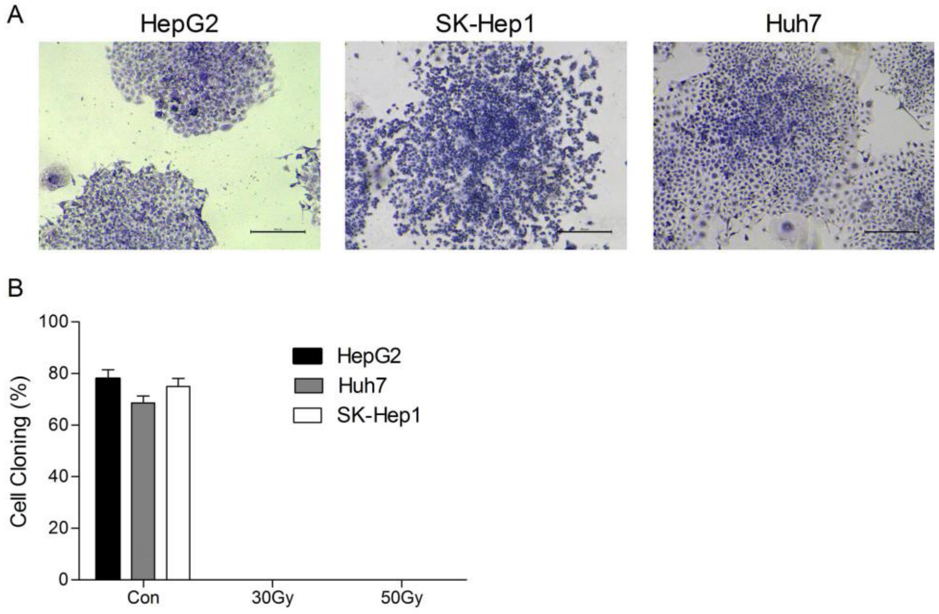

Supplementary Figure 1. X-ray irradiation inhibits the colony formation in tumor cell lines *in vitro*. After X-ray irradiation, HepG2, Huh7, and SK-Hep1 cells separated were cultured for 14 d. Representative Giemsa staining (scale bar = 100 μm) in 0 Gy X-ray irradiated HepG2, Huh7, and SK-Hep1 cells after culturing for 7 d (A). Colony formation rates in X-ray irradiated tumor cells after culturing for 7 d were detected (B). Data are means ± SEM; n = 6.

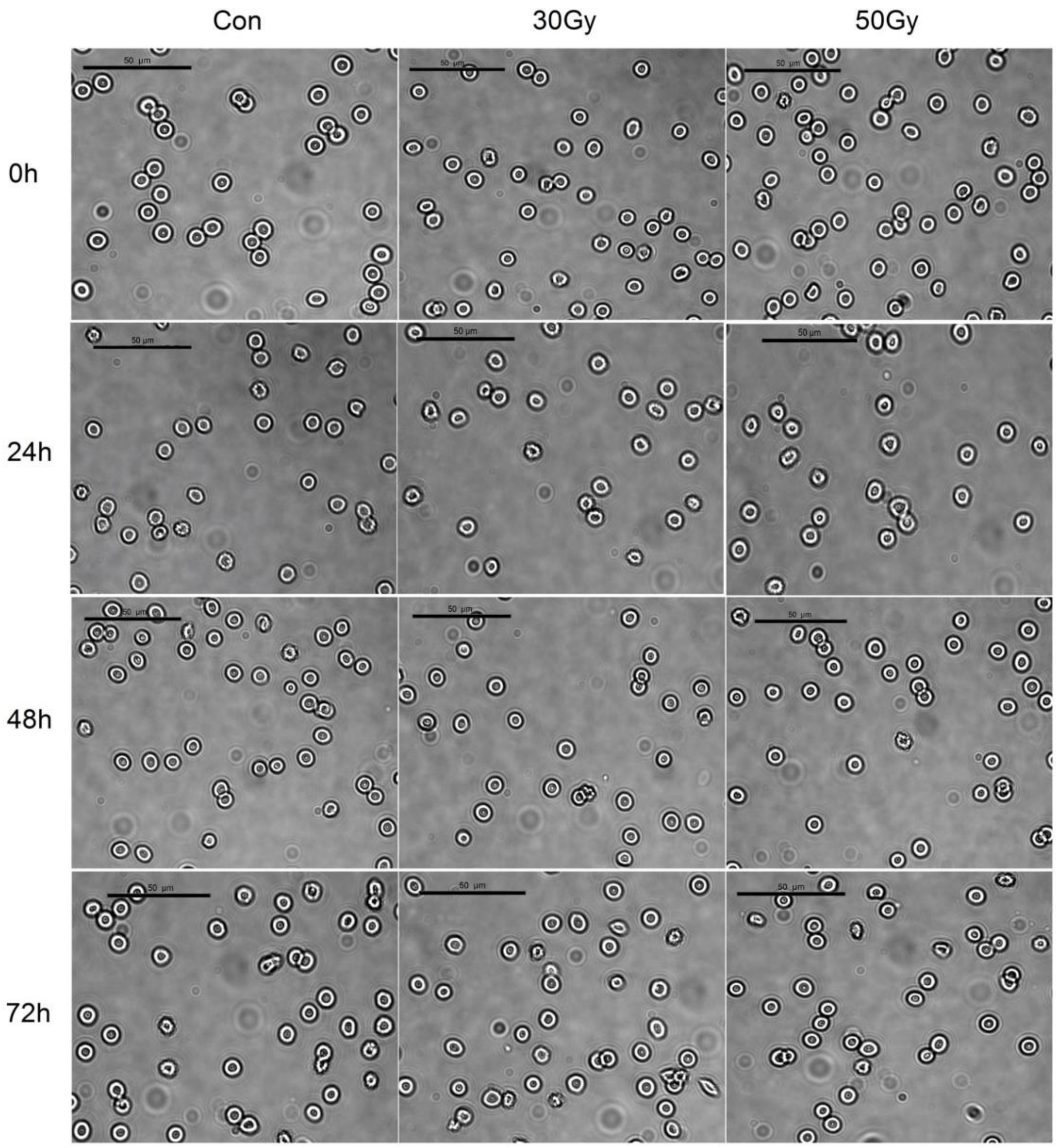

Supplementary Figure 2. Effects of X-ray irradiation on the morphology of erythrocytes *in vitro*. After X-ray irradiation, erythrocytes were cultured for 0, 24, 48 and 72 hours. Representative morphology of erythrocytes (scale bar = 50 μm) were detected under an inverted microscopy.
